# Characterization of a female germline and early zygote promoter from the transcription factor bZip1 in the dengue mosquito *Aedes aegypti*

**DOI:** 10.1186/s13071-020-04216-w

**Published:** 2020-07-17

**Authors:** Bianca B. Kojin, James K. Biedler, Zhijian Tu, Zach N. Adelman

**Affiliations:** 1 Department of Entomology and Agrilife Research, Texas A&M University, College Station, TX USA; 2 Department of Biochemistry and the Fralin Life Sciences Institute, Virginia Tech, Blacksburg, VA USA

**Keywords:** Mosquito, Promoter, Regulatory sequence, Germline, Zygote, Maternal expression, Ovaries, Embryo

## Abstract

**Background:**

The wide distribution of *Aedes aegypti*, the main vector of dengue and yellow fever viruses, currently puts three billion people in the world at risk of infection with these viruses. Continuous transmission of these and other viruses despite aggressive efforts to prevent this emphasizes the need to develop new control strategies. Proposals to control disease transmission based on vector engineering, including both population suppression and population replacement, rely on the development of transgenes under the control of regulatory elements able to drive molecules in a specific tissue, time and strength.

**Methods:**

Here we report the characterization of a promoter active in both the female germline and early zygote, derived from the transcription factor bZip1 in the mosquito *Ae. aegypti*, using transposon-based methods and RT-qPCR.

**Results:**

We generated seven transgenic lines carrying *AabZip1*-reporter constructs and observed expression in both the ovary and early embryo. RT-qPCR analysis was performed to evaluate transcript expression patterns for each line, confirming that transgenic expression from the *AabZip1* promoter largely recapitulated the endogenous expression pattern, albeit the strength of maternal expression appeared to be strongly influenced by chromosomal position.

**Conclusions:**

This study provides a new regulatory sequence that can be useful for generating transgenic lines that can become a tool in vector control strategies.
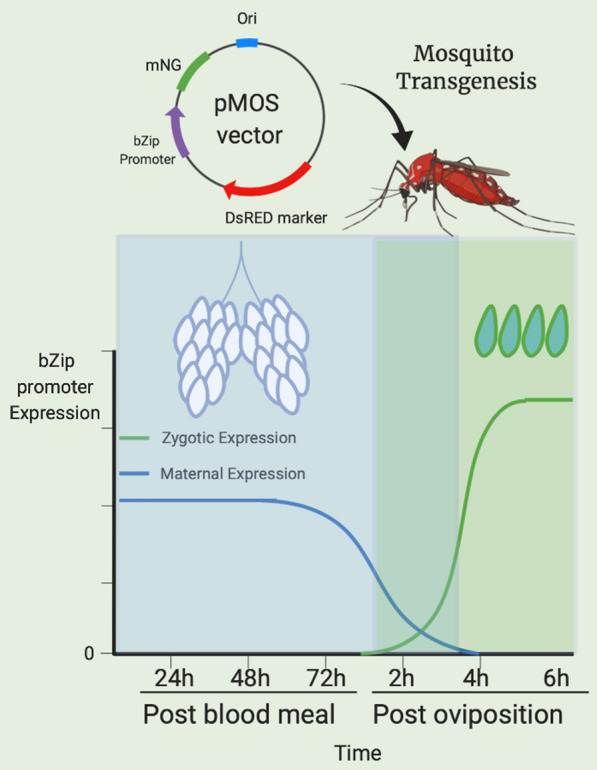

## Background

*Aedes aegypti* is the main vector for arboviruses including dengue, yellow fever, chikungunya and Zika viruses. All together, these pathogens are responsible for hundreds of millions of infections and put 40% of the world population at risk [1–6]. Factors like anthropophilia and adaptation to urban environments contribute to the success of *Ae. aegypti* as a vector for so many diseases [7]. Mosquito population replacement and population suppression approaches based on genetic modification are currently being developed to restrict the spread of vector-borne diseases. The concept for population replacement is based on the hypothesis in which the natural pathogen-susceptible mosquito population is replaced by a refractory one, decreasing or eliminating the transmission of that pathogen to humans [8], whereas population suppression involves the release of altered mosquitoes that can cause elimination or reduction of the population, impacting disease transmission [9]. In either approach, functional characterization of regulatory sequences capable of driving transgene expression with a desired spatial and temporal pattern is a prerequisite. For example, promoter sequences driving maternal or zygotic expression are essential for the development of gene drive approaches based on maternal-effect dominant embryonic arrest (Medea) or homing-based drive, which are an essential part of the population replacement strategy [10–12]. Such promoters may also be useful for manipulating sex ratios as sex determination occurs early during development which can be used for suppression strategies.

In *Ae. aegypti*, NIX is a dominant male determining factor. Somatic knockout of the *Nix* gene results in feminized genetic males while ectopic expression of Nix results in the opposite, with genetic females displaying nearly complete male genitalia [13]. Once Nix is stably expressed in transgenic lines, the conversion is complete, with males that are fertile (with assisted mating) and unable to blood-feed [14]. The Tetracycline Inducible Expression system, or Tet-off system, offers the possibility of controlling ectopic gene expression. This system relies on tetracycline or its analogs to induce or repress gene expression; in the absence of tetracycline (TET), the transactivator (tTA) binds to a Tet response element (TRE) inducing gene expression. However, in the presence of TET, tTA binds with higher affinity to the antibiotic, reducing gene expression [15]. Early embryonic and maternal conditional expression of Nix using the Tet-off system [15] could be an approach to skew sex ratios towards males for sterile-insect technique programmes, while offering the possibility also to produce both sexes in order to maintain the transgenic colony. That is particularly interesting in a factory setting for mass release purposes aiming at vector control, as the mass production of both sexes represents a challenge. Physical separation of males for release from females is typically feasible only after pupation, thus requiring capacity and resources to rear unneeded females throughout larval development. However, females are essential for colony maintenance as mentioned above [16]; therefore, a system that allows the switch between male only and both sexes production, would contribute to the success of this type of vector control strategy.

RNAseq experiments [17–21] or gene ontology searches based on genes characterized in other organisms like *Drosophila melanogaster*, have provided data for identification of candidate regulatory sequences in mosquitoes. While a number of promoters active in the *Ae. aegypti* female germline or early zygote have been characterized [18, 22–24], it is not clear if these candidates provide an expression pattern suitable for the ectopic expression of a sex-determining gene such as *Nix*. For example, maternal activity too early in female germline development might result in sterility, while insufficient zygotic activity might result in only partial sex conversion.

Here we describe the ability of the regulatory sequence derived from the *Ae. aegypti bZip1* gene to drive the expression of transgenes. *AabZip1* is maternally expressed (late in ovarian development) and deposited in embryos and is also zygotically expressed. We show that the regulatory sequence located upstream of *AabZip1* can drive the expression of a fluorescent marker in transgenic mosquitoes in a manner that largely replicates the endogenous gene expression pattern. Unexpectedly, when the fluorescent marker was replaced by the transcription factor tTA, the transgenic lines obtained not only lacked the maternal expression, but also displayed restricted zygotic mRNA accumulation. The implications of these findings are further discussed.

## Methods

### RNAseq data analysis

RNA-seq data from Akbari et al. [19] that had been re-mapped to genome version AaegL5.0 were downloaded from the VectorBase FTP server (ftp://ftp.vectorbase.org/public_data/bam/aedes_aegypti/) and counts per gene determined using featureCounts [25]. Raw counts were linear normalized based on transcript length and library size to obtain fragments per kilobase per million reads (FKPM) data for each gene. FKPM data were log_10_-transformed using the following formula to avoid negative values [log_10_^FKPM^ = log_10_ (1 + FKPM)]. Transformed data were visualized using Morpheus (https://software.broadinstitute.org/Morpheus).

### Donor plasmid assembly

Donor plasmids were constructed for *mariner* transposable element-mediated transformation, using the terminal inverted repeats of the *mariner Mos1* element flanking the transgenic cargo [26]. An approximately 4.6 kb sequence upstream of the coding sequence from the *Ae. aegypti bZip1* gene AAEL009263 (Vectorbase.org), that includes the first exon and intron, was used as the promoter to drive expression of the transgene(s). The first exon and intron were included in order to increase gene expression by affecting transcription rate, stability and nuclear export [27]. The transgenes terminated with the predicted 3’ UTR from the *Anopheles stephensi* β2 tubulin gene ASTEI09889 (Vectorbase.org). A total of 150 bp genomic sequence 3’ of the stop codon was used, including 83 bp downstream from the polyA signal. Three separate constructs encoding the following transgenes were generated: mNeonGreen fluorescent protein (mNG) [28]; tTA trans activator protein [15]; tTA and mNG, using the T2A sequence between their ORFs. The T2A small peptide (~ 18 aa) was used to enable multicistronic expression [29]. The DsRed fluorescent protein [30] was used as the transgenic marker, driven by the *Ae. aegypti* polyubiquitin promoter [31]. Transgenes were synthesized and cloned into the *Mos1* backbone plasmid [22] by Epoch Life Science, Inc. (Missouri City, TX, USA).

### Mosquito rearing and transformation

*Aedes aegypti* Liverpool strain (LVP) was used for all experiments and were kept confined in chambers in an insectary at 28 °C, 60–70% humidity and 14:10 h light:dark cycle. The colony was maintained, and experiments were performed exclusively on defibrinated sheep blood (Colorado Serum Company, Denver, CO, USA) using an artificial feeding system. The generation of *Ae. aegypti* transgenic lines was performed as described previously [26]. Briefly, preblastoderm embryos were microinjected with a mixture of one of the donor plasmids (bZip-mNG, bZip-tTA or bZip-tTA-mNG) at 0.5 μg/μl and the helper plasmid (pGL3-pUbMos) [31, 32] at 0.3 μg/μl using FemtoJet® equipment (Eppendorf, Hamburg, Germany) and pulled borosilicate glass capillaries (World Precision Instruments, Sarasota, USA). Females from surviving injected embryos were pooled into 20–25 individuals per cage and backcrossed to LVP in a 1:1 ratio, while males were mated with LVP females in a 1:5–10 ratio. The G_1_ progeny from injected embryos were screened for DsRed of the marker gene during larval stages. Transgenic lines obtained from female and male pools were identified by the letter P and F, respectively, followed by a number representing the cage they came from (e.g. bZip-mNG P3 or bZip-mNG F1). All experiments were performed with heterozygous mosquitoes.

### Inverse PCR

Genomic DNA was extracted from the transgenic mosquitoes using the NucleoSpin® Tissue Kit (Macherey-Nagel, Duren, Germany) and digested overnight with Sau3AI and CviAII. The digested DNA was purified and ligated in the presence of excess T4 ligase (New England Biolabs, Ipswich, USA) and used as a template for the following PCR reaction: 98 °C for 3 min., 29 cycles of 98 °C for 1 min, 65 °C for 45 s, 72 °C for 1 min, and 1 cycle of 72 °C for 5 min, using the primers listed in Additional file 1: Table S1. Amplification products were gel-extracted and sequenced. VectorBase (http://www.vectorbase.org) [33] was searched for sequences corresponding to the junctions between transposon landing sites on *Ae. aegypti* genome and transposon arms using the BLASTn tool.

### Tissue collection

Females from transgenic and LVP mosquito strains were blood-fed and ovaries were dissected in phosphate buffer saline (PBS) at 24, 48 and 72 h post-blood-meal (PBM); similar dissections were performed for non-blood-fed females (NBF). For tissue specificity experiments, “carcass” refers to females after ovaries were removed at 72 h PBM; “head and thorax” (H/T) refers to the head and thorax of females not including the abdomen at 72 h PBM. For embryo collection, females from transgenic and LVP controls were blood-fed and after 72 h they were placed in tubes with access to wet cotton wool and a disk of filtered paper on top and allowed to lay eggs for 20 min. After that, females were released into cages and embryos collected.

### Imaging

To determine the pattern of DsRed and mNG expression in larvae, freshly dissected ovaries, and embryos, tissues were placed on a glass slide in PBS and immediately visualized using a Leica M165 FC stereomicroscope (Leica, Wetzlar, Germany) equipped with filters for fluorescence: 49002 ET-GFP (excitation, 470/40; emission, 525/50; dichroic, 495) and 49005 ET-DsRed (excitation, 545/30; emission, 620/60; dichroic, 570). Fluorescence and bright-field images were acquired using AmScope (version x64, 3.7.58492015) software with the same settings throughout the experiment.

### RT-qPCR

Total RNA was extracted using TRIzol (Thermo Fisher Scientific, Waltham, USA) following the manufacturer’s protocol. The isolated RNA was treated with ezDNAse (Thermo Fisher Scientific, Waltham, USA) and quantified on a SpectraMax spectrophotometer (Molecular Devices, Sunnyvale, USA). One microgram of treated RNA was used to synthesize cDNA using SuperScript IV VILO Master Mix (Thermo Fisher Scientific, Waltham, USA) that contains both oligo (dT)18 and random hexamer primers also following the manufacturer’s protocol. All experiments were performed in biological duplicates with five mosquitoes per sample, except for the experiment analyzing *bZip1* and mNG expression on dissected tissues, which was performed in biological triplicate.

RT-qPCR was carried out with SsoAdvance Universal SYBR Green Supermix (BioRad, Hercules, USA) on a CFX69 Touch Real-Time PCR Detection System (BioRad, Hercules, USA). The primers used were designed on Primer 3 server (v.0.4.0) [34, 35] with the amplification products not longer than 135 bp. Amplification efficiency was verified to be 0.9–1.0 in a reaction using cDNA (bZip1 primers) or DNA plasmid (mNG and tTA primers) as a template. Reactions were performed with 1:50 diluted cDNA in technical triplicates, with the primers listed in Additional file 1: Table S1, following the cycling parameters: 30 s at 95 °C, 45 cycles of 15 s at 95 °C, 15 s at 60 °C and 10 s at 72 °C, and melt curve analyses at 65–95 °C. The dCT method was used to calculate expression relative to the rpS7 gene [36].

## Results

### Selection of *bZip1* as a source of a novel maternal/early zygotic promoter

An examination of *Ae. aegypti* early zygotic transcriptomes aimed to study the activation and expression of early zygotic genes [17], led to the identification of a gene whose RNA transcript was both maternally deposited and zygotically transcribed. The predicted protein encoded by this gene contains a single basic leucine zipper domain and thus is anticipated to be a transcription factor; we refer to this gene as *AabZip1*. *AabZip1* was found to have clear 1:1 orthologs with genes in *Culex* and *Anopheles* mosquitoes, but no identifiable ortholog in *D. melanogaster* (Additional file 1: Table S2). Interestingly, distant orthologs were identified in *Ctenocephalides felis* (cat flea) and *Clunio marinus* (non-biting midge) (Additional file 1: Table S3). A phylogenetic tree of AabZip1 protein and its closest orthologs shows this in more detail (see Additional file 1: Figure S1).

**Fig. 1:**
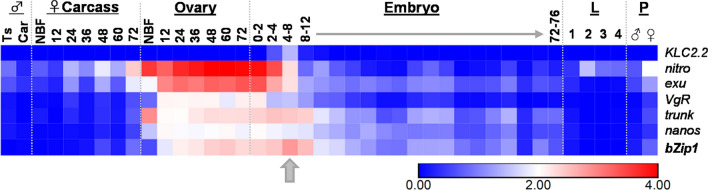
RNA-seq transcriptional profile of maternal/zygotic genes with regulatory sequences used to control transgene expression in *Ae. aegypti*. RNA-seq data derived from adult males (Ts, testes; Car, carcass), adult female carcass or ovaries (NBF, non-blood-fed; 12–72 h after blood-feeding), embryos (0–76 h-old), larvae (stages L1–L4) and pupae (male and female). Block arrow indicates the peak of *bZip1* expression in 4–8 h-old embryos. Raw data are from Akbari et al. [19]. Scale is log_10_ (FPKM+1)

To compare the expression pattern of *AabZip1* with those of other genes whose regulatory sequences have already been utilized to control transgene expression, we mined the *Ae. aegypti* developmental transcriptome [19] for *AabZip1*, *nanos*, *exu*, *nitro*, *trunk*, *VgR* and *KLC2.2* [19, 37–39]. Consistent with its original identification as any early zygotic gene [17], *AabZip1* was most abundantly expressed in 4–8 h embryos (Fig. 1). In contrast to the kinesin light chain gene (*KLC2.2*) which was expressed specifically at this time point, *AabZip1* was also transcribed in the ovary specifically in response to a blood-meal. This was not the case for *exu*, *nitro* and *trunk* (and to a lesser extent *nanos*), where robust expression occurred in the ovary even in the absence of a blood-meal. From these data, we conclude that *AabZip1* has a unique expression pattern that may be of value in controlling the expression of a transgene.

### *bZip1* promoter yields expression similar to the endogenous *bZip1* gene

**Fig. 2:**
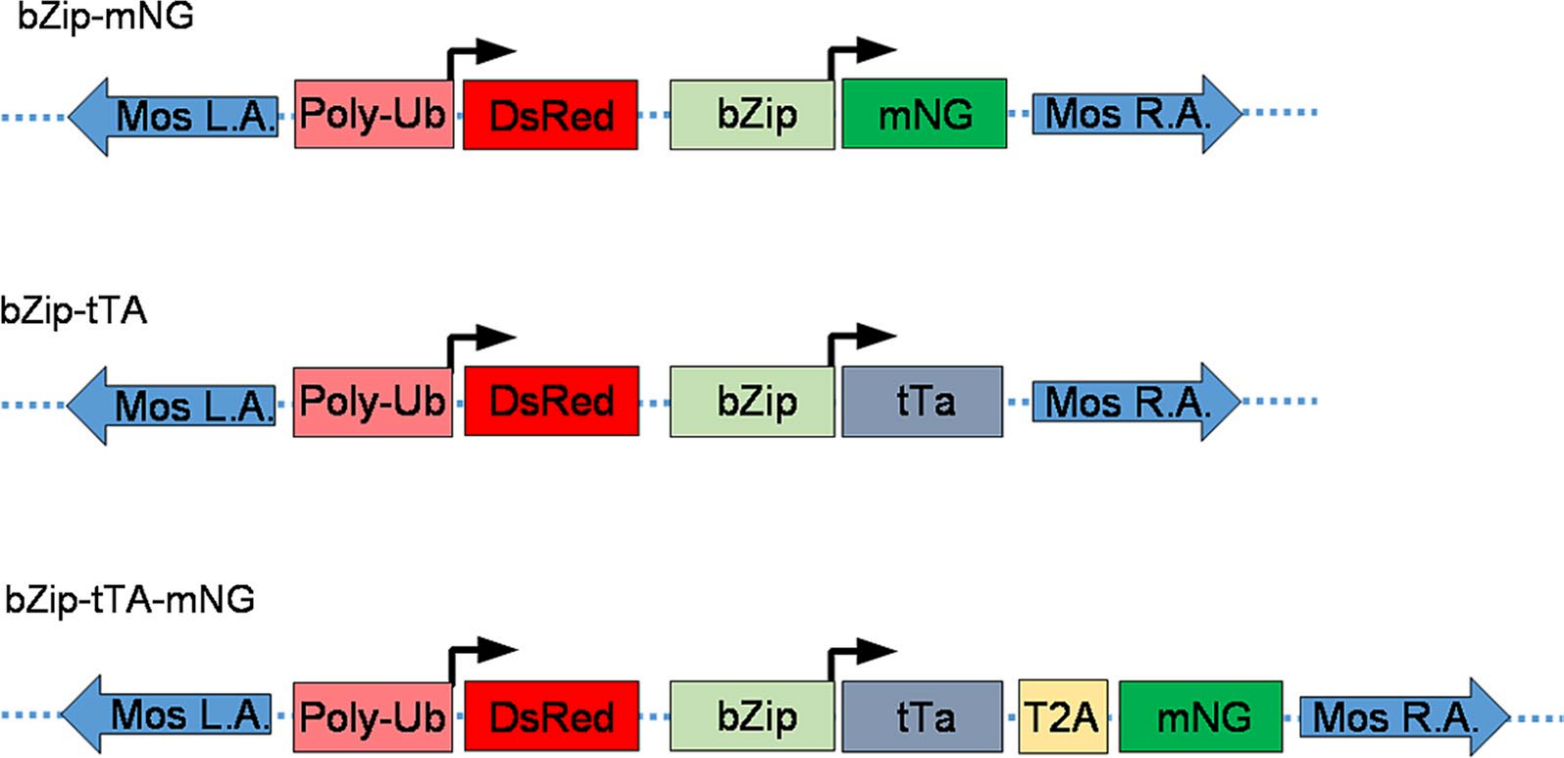
Schematic representation of the transformation constructs. All constructs consisted of the mariner MosI right arm (Mos R. A.) and left arm (Mos L. A.) and carried the marker gene cassette PolyUbDsRed. The polyubiquitin promoter is expected to express DsRED in all stages of mosquito development and most tissues. The dotted lines represent sequence from the plasmid backbone. The bZip-mNG construct encodes a gene cassette composed of the *bZip1* promoter driving the expression of the mNeon Green fluorescent protein (mNG). bZip-tTA and bZip-tTA-mNG constructs also carry a gene composed of the *bZip1* promoter region, but driving the expression of the transactivator (tTA) from the Tet-off system; construct bZip-tTA-mNG expresses along with the tTA, the self-cleaving peptide T2A coupled with the mNG fluorescent protein

**Fig. 3:**
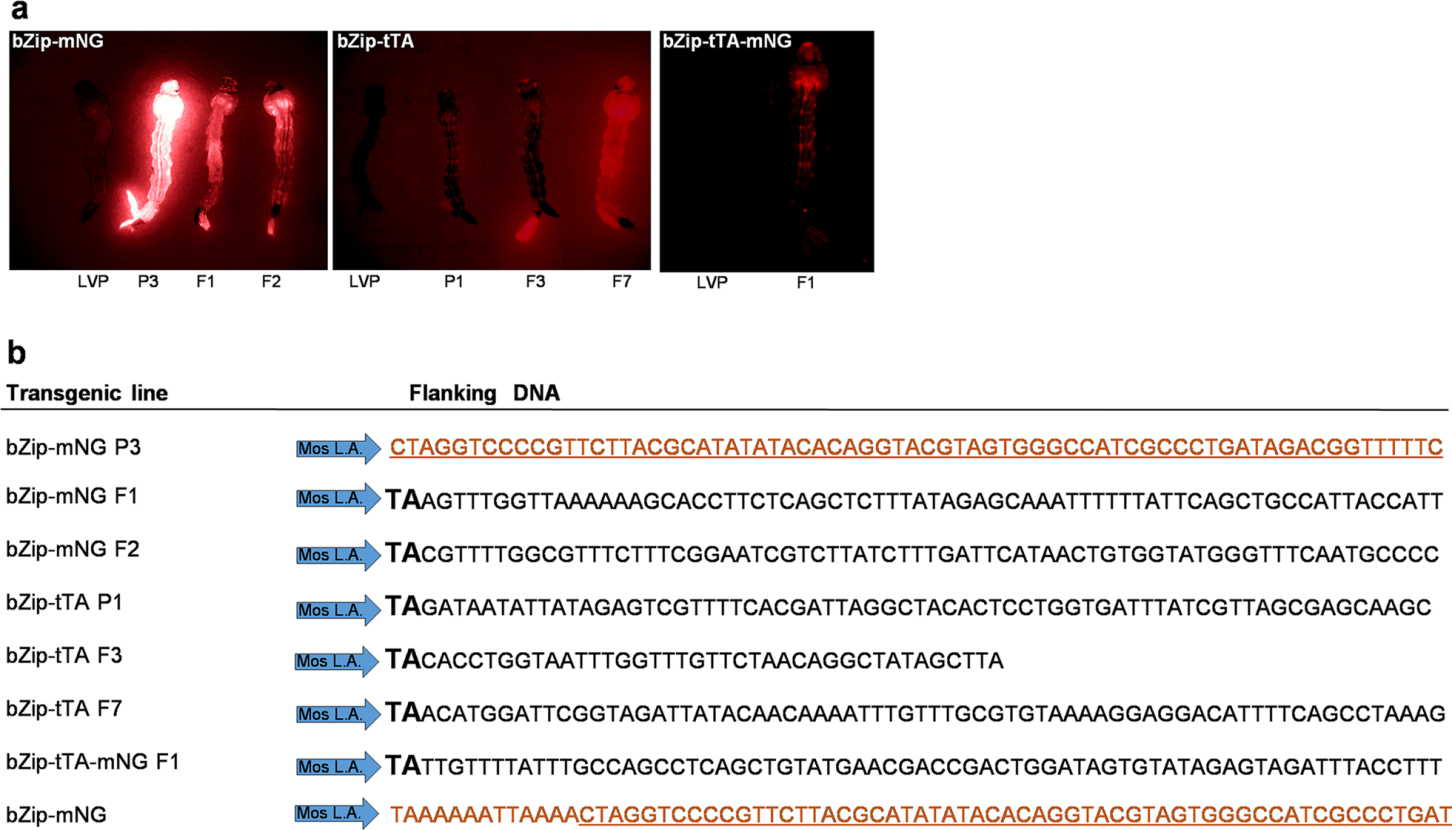
Establishment of *bZip1* transgenic lines. **a** Expression of DsRED in larvae of each transgenic line compared to the LVP control. **b** DNA sequence obtained by inverse PCR flanking the transposon random insertions from bZip1 transgenic constructs. TA dinucleotide is indicated in bold; in black, *Ae. aegypti* genomic DNA; in orange, plasmid sequence; and underlined, the identical sequence shared between the plasmid (bZip-mNG) and the sequence obtained by inverse PCR from line bZip-mNG P3 flanking transposon left arm. P and F after each transgenic line denotes that the line was generated from a female G#1 pool or male G#1 pool, respectively and the number after both letters, the cage identification on the G#1 cross

We sought to evaluate if a genomic fragment (4587 bp) upstream of the open reading frame (ORF) of the *AabZip1* gene was able to drive the expression of a heterologous gene in a manner that recapitulated the endogenous expression of this gene. To this end, a donor vector (bZip-mNG) containing the putative promoter region controlling the expression of a fluorescent protein (Fig. 2), along with the pGL3 polyUbMos helper plasmid, were injected into 2126 preblastoderm *Ae. aegypti* embryos. From those embryos, 415 individuals reached adulthood and were outcrossed to generate three independent transgenic lines (Table 1). As expected, the marker DsRed could be observed in all three transgenic lines throughout the body in larval stages and in the somatic tissues of the ovaries indicating the presence of the transgene. The intensity of DsRed expression differed markedly between the three lines, with the highest expression occurring in line bZip-mNG P3 (Fig. 3a), potentially due to position effects of the surrounding chromatin and/or different copy number of the transposon. To confirm transgene insertion into the chromosome, inverse PCR was performed and the sequences flanking the transposable element arms were determined (Fig. 3b). The sequences obtained from transgenic lines bZip-mNG F1 and bZip-mNG F2 were accompanied by the duplication of the dinucleotide TA, normally occurring with the pMos1 transposable element genomic insertions [26]. In addition, the sequences matched the *Ae. aegypti* genome using the BLASTn tool, indicating the insertion in a cut-and-paste manner. However, the precise location of the genomic insertion from both lines could not be identified, as multiple matches with high identity ranging between 99.7–99.3% in bZip-mNG F1 and 93.3–94% in the bZip-mNG F2 line were retrieved, possibly due to the highly repetitive regions in *Ae. aegypti* genome. We also found that inverse PCR was only reliable to identify the DNA flanking the pMOS transposable element left arm as we only identified sequences amplified from it and not the right arm; this contributed to our inability to identify the precise landing spot of each insertion. As for transgenic line bZip-mNG P3, only plasmid junctions were obtained despite multiple attempts with different restriction enzymes, suggesting a non-canonical, potentially complex insertion (Fig. 3b).

**Table 1:** Microinjection results of bZip-mNG, bZip-tTA and bZip-tTA-mNG constructs

Construct	No. of embryos injected	Larvae hatching (%)^a^	Survival (%)	No. of screened larvae^b^	No. of DsRed positive larvae	No. of transgenic lines	Transformation rate (%)^c^
bZip-mNG	2126	424 (20)	415 (19)	20,484	104	3	1.4
bZip-tTA	1825	634 (35)	634 (35)	30,365	14	3	0.9
bZip-tTA-mNG	2065	399 (19)	370 (18)	50,518	50	1	0.5

^a^Percentage of injected embryos that hatched into L3-L4 larvae

^**b**^Percentage of injected embryos that hatched into adults

^c^Percentage of independent transformed lines generated per fertile adult (assumes ~ 50% fertility)

**Fig. 4:**
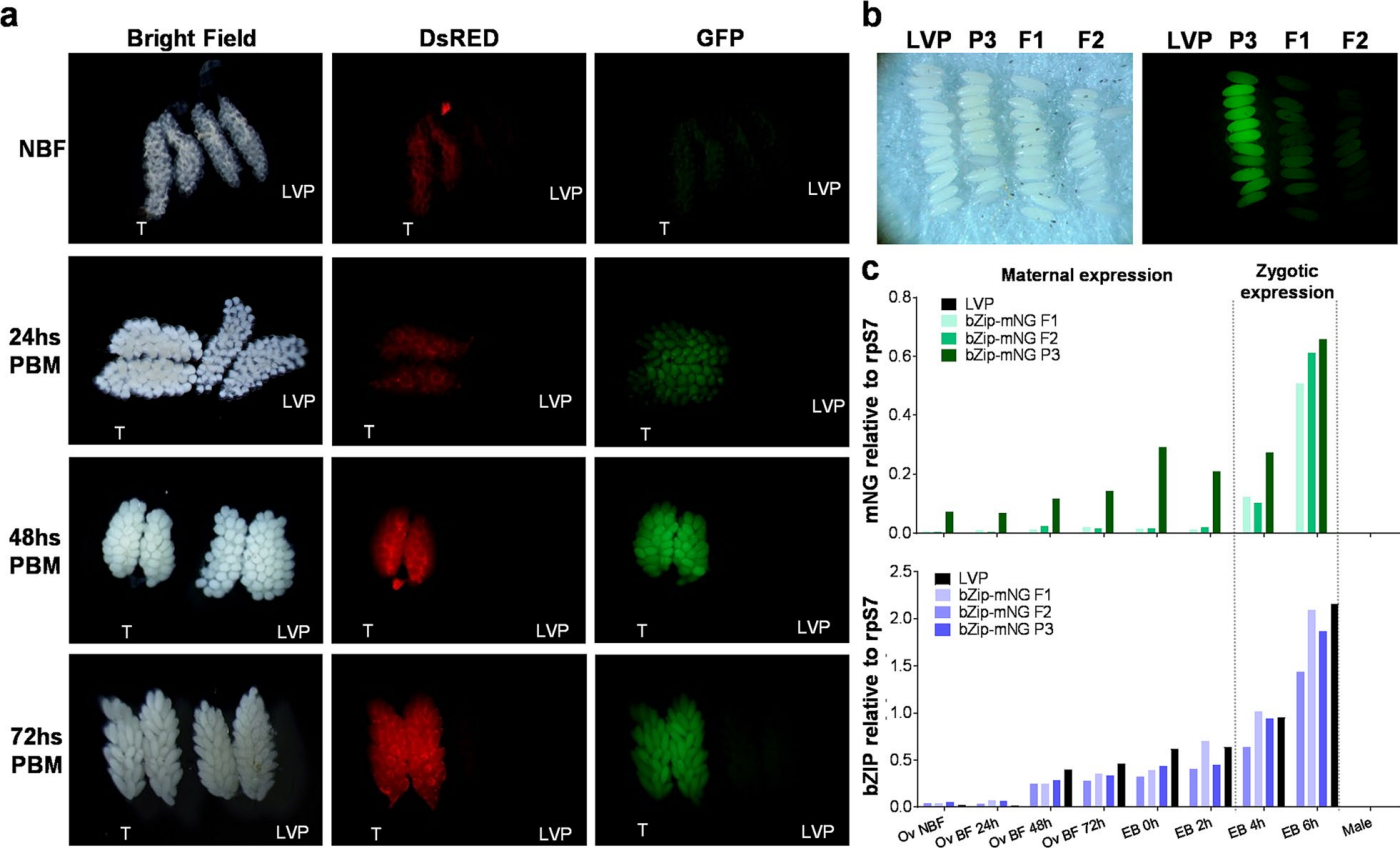
Expression profile of mNG in ovaries and embryos of bZip-mNG transgenic mosquitoes. **a** Ovaries from bZip-mNG P3 transgenic line (T) and wild type control (LVP) of NBF and 24, 48 and 72 h PBM females. **b** Accumulation of mNG in embryos of bZip-mNG P3, F1 and F2 transgenic lines at 0 h after they were laid. **c** Quantification of accumulated mNG and endogenous *bZip1* transcripts by RT-qPCR in ovaries dissected from transgenic bZip-mNG P3, F1 and F2 and LVP females NBF, 24, 48 and 72 h post-blood-meal and embryos 0, 2, 4 and 6 h after being laid

To determine if the *AabZip1* genomic fragment contained the appropriate cis sequences important for maternal expression, ovaries from 24, 48 and 72 h post-blood-meal and non-blood-fed (NBF) females were dissected and analyzed for mNG expression. Different intensities of mNG expression between 24 and 72 h were observed between the three transgenic lines, where bZip-mNG P3 showed expression at 24 h, peaking at 48 h and remaining the same until 72 h (Fig. 4a). No apparent difference in the intensity could be observed at the three time points for bZip-mNG F1, as the expression remained stable throughout those time points (Additional file 1: Figure S2a), while bZip-mNG F2 showed a peak mNG intensity at 48 h, similar to that observed in line bZip-mNG P3 (Additional file 1: Figure S2b).

Visualization of mNG in freshly laid eggs (Fig. 4b) and mNG transcript accumulation in embryos 0 and 2 h after being laid detected by RT-qPCR (Fig. 4c) confirmed the maternal deposition of protein and RNA. Accordingly, mNG transcript accumulation in embryos from 4 and 6 h after being laid (Fig. 4c) confirmed zygotic expression. Although the temporal expression of the transgene followed the same pattern as the endogenous *AabZip1*, the intensity of expression on all three lines was approximately 3 times lower than the *AabZip1* gene expression (Fig. 4c). Similar to endogenous *AabZip1* expression, no accumulation of the transgenic transcript could be detected in adult males (Fig. 4c).

**Fig. 5:**
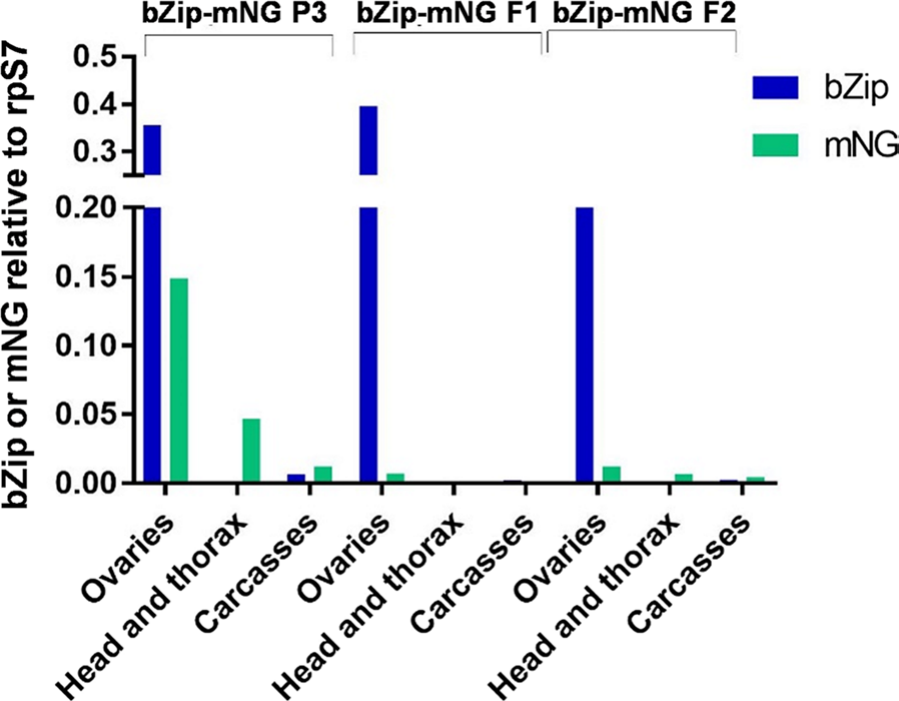
Tissue specificity of *AabZip1* promoter expression. Quantification of mNG transcript by RT-qPCR from RNA isolated from ovaries, head and thorax (H/T) and carcasses for bZip-mNG P3, F1 and F2 transgenic females and LVP at 72 h PBM

The spatial expression of the transgene was analyzed in the ovaries, carcasses and head and thorax of each transgenic line. Transcript accumulation of the transgene was observed in the ovaries in all lines, similar to the endogenous *AabZip1* gene. Lines bZip-mNG P3 and bZip-mNG F2 also showed low-level expression in head and thorax and carcasses; this was not the case for bZip-mNG F1, where expression was confined to the ovaries (Fig. 5). The expression in the heads and thorax only of the mNG and not bZip1 in lines bZip-mNG P3 and bZip-mNG F2 may denote a lack of one or more critical regulatory elements in the promoter, or the susceptibility of the transgene cassette to local position effects. This should be considered for any future application using this promoter fragment.

### Failure of *bZip1* promoter to provide robust expression of transgenic tTA

In order to evaluate the ability of the *AabZip1* promoter to drive the expression of the tTA effector molecule, the donor plasmid was modified in two different ways. In bZip-tTA, the mNG sequence was replaced by tTA (Fig. 2), while in bZip-tTA-mNG, the tTA sequence is followed by the self-cleaving peptide T2A and mNG ORF (Fig. 2).

Each donor plasmid was mixed with the pGL3 polyUb Mos helper to generate new transgenic lines, with bZip-tTA injected into 1825 embryos and bZip-tTA-mNG injected into 2065 embryos, resulting in three and one transgenic line, respectively (Table 1). Inverse PCR was performed with genomic DNA extracted from the four transgenic lines, all of them contained the dinucleotide duplication signature from pMos1 element insertion and all the sequences matched the *Ae. aegypti* genome using BLASTn tool (Fig. 3b). Once again, the precise chromosome location of the insertion from these lines could not be retrieved, as several matches with high identity were found, ranging between 98.5–100% in bZip-tTA P1, 80.5–92.3% in bZip-tTA F3, 97.9–100% in bZip-tTA F7 and 93.6–98.2% in bZip-tTA-mNG F1.

**Fig. 6:**
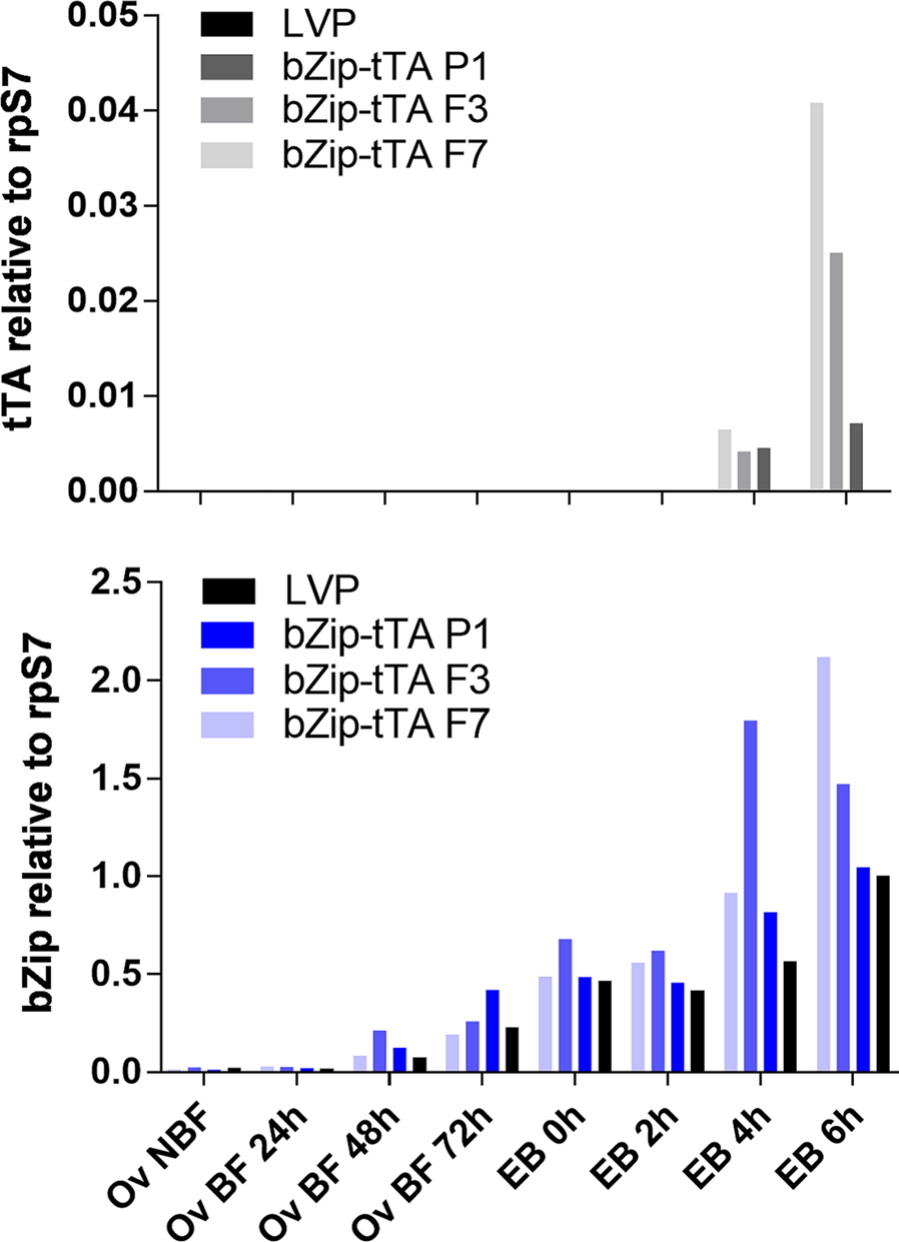
tTA abundance from bZip-tTA transgenic lines. Quantification of mRNA accumulation of tTA and endogenous *AabZip1*was determined by RT-qPCR of ovaries dissected from transgenic bZip-tTA P1, F3, F7 transgenic lines and LVP at 24, 48 and 72 h post-blood-meal and embryos 0, 2, 4 and 6 h after being laid

Expression profile by RT-qPCR performed on the three bZip-tTA lines revealed no evidence for maternal expression (Fig. 6). Expression was detected in embryos 4 h and 6 h after being laid, although at a level that was approximately 27 times lower than the mNG gene expression in bZip-mNG lines (Fig. 6).

**Fig. 7:**
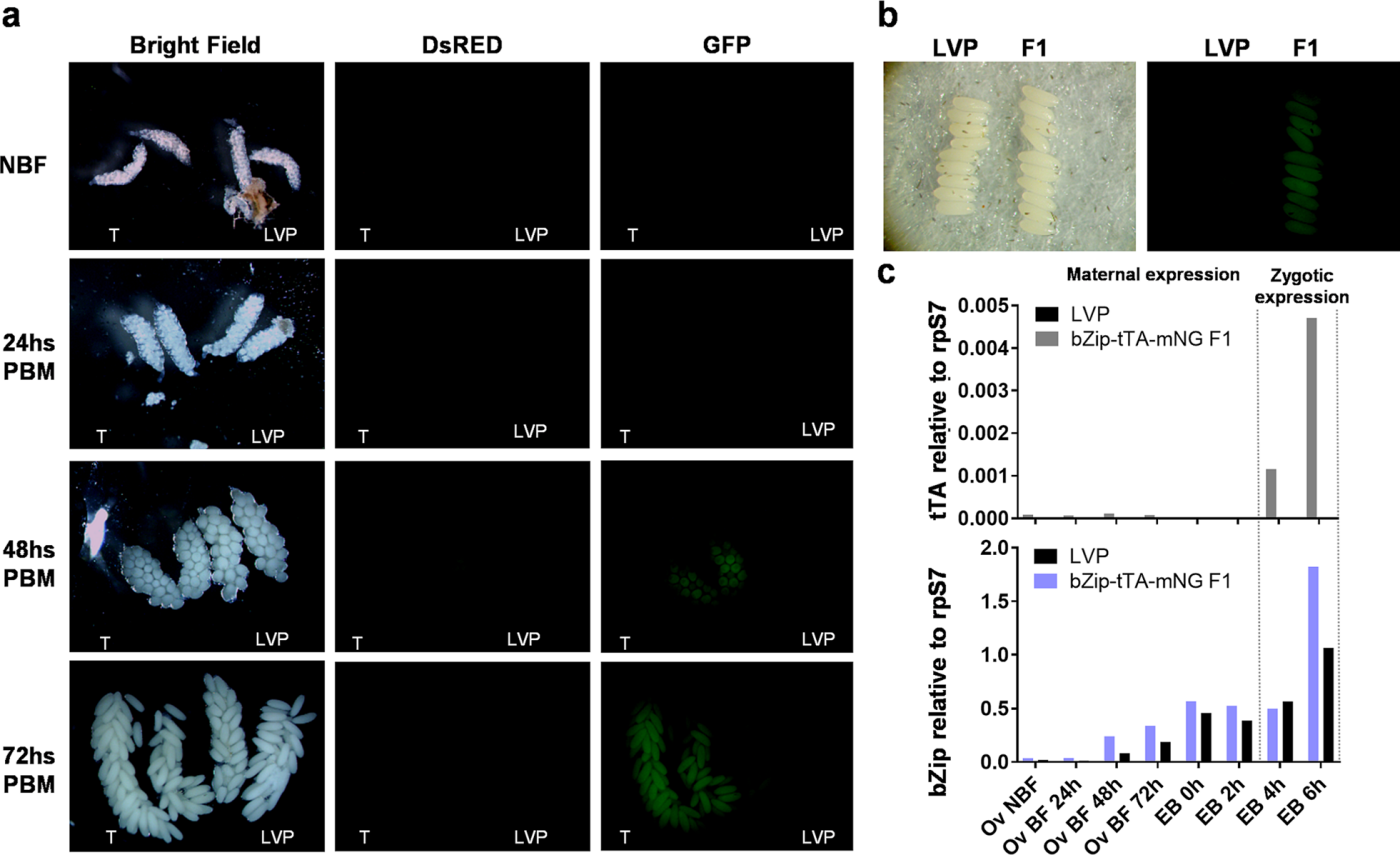
Expression profile of tTA in ovaries and embryos of bZip-tTA-mNG transgenic mosquitoes. **a** Ovaries from bZip-tTA-mNG F1 transgenic line (T) and wild type (LVP) of non-blood-fed females (NBF) and females 24, 48 and 72 h after a blood meal. **b** Accumulation of mNG in embryos of bZip-tTA-mNG F1 transgenic line at 0 h after they were laid. **c** Quantification of accumulated tTA and endogenous *AabZip1* transcripts by RT-qPCR in ovaries dissected from transgenic bZip-tTA F1 and LVP females NBF, 24, 48 and 72 h post-blood-meal and embryos 0, 2, 4 and 6 h after being laid

We next analyzed the transgenic line bZip-tTA-mNG F1. This line differs from the previous one as it contains the mNG fluorescent marker being expressed along with the tTA to assist with expression tracking. The fluorescent protein was detectable in ovaries dissected at 48 h and 72 h PBM, but not in those dissected at 24 h PBM, nor in NBF mosquitoes (Fig. 7a). This differs from the previous bZip-mNG lines as the protein accumulation was visualized starting at 24 h PBM and in higher intensity as well. Maternal deposition of the protein was confirmed in embryos freshly laid (Fig. 7b). Another important difference between bZip-mNG and bZip-tTA-mNG F1 lines is the expression of the marker gene. In the later one, DsRed could not be detected in ovaries dissected at any time points, whereas in bZip-mNG lines DsRED was present in all of them.

The RT-qPCR data indicated low transcript accumulation in ovaries at all time points collected (Fig. 7c), as the intensity of expression in this line was approximately 1800 times lower than the endogenous *AabZip1*. In embryos, expression of the tTA transcript could not be detected at 0 and 2 h after being laid, indicating a lack of maternal RNA deposition. In contrast, tTA transcripts were detected at 4 and 6 h-old embryos, indicating some level of zygotic transcription.

## Discussion

Transgene-based vector control strategies rely on a well-developed understanding of the molecular biology of regulatory factors used to express proteins in a specified tissue and developmental time. To date, a number of genes and their regulatory sequences have been characterized that display maternal and zygotic expression in mosquitoes, important features for driving the expression of proteins in tissues like ovaries and also the embryo. Those regulatory sequences are of special interest for genetic strategies like population suppression and population replacement. The present study evaluated the potential use of a regulatory sequence derived from upstream of the gene *bZip1* in transgenic *Ae. aegypti*. *AabZip1* is an ortholog to the *An. stephensi bZip1* gene first identified by RNA-seq of early ovary-specific genes [20]. It contains a basic leucine zipper domain and is conserved among mosquitoes including species in the genera *Aedes*, *Culex* and *Anopheles.* The 4587 bp upstream the *AabZip1* open reading frame was used in initial experiments to drive the expression of a fluorescent protein in transgenic *Ae. aegypti* for promoter characterization. In all three bZip-mNG lines obtained, the transgene largely mirrored the zygotic component of expression of the endogenous *AabZip1* but not the intensity, as the expression in bZip-mNG lines were lower in comparison to the endogenous gene. Ovary-specific transcript expression was detected in NBF until 72 h PBM ovaries as well as translation, though maternally controlled expression was much more variable between transgene insertion sites. The presence of the fluorescent protein in 0 h embryos, and transcripts in 0–2 h embryos, demonstrates deposition of mRNA/protein from the mothers, as zygotic transcription is not expected to start in embryos before 2 h post-oviposition [19]. The promoter was also able to induce zygotic expression in all bZip-mNG lines obtained, as transcript accumulation was detected from embryos 4–6 h post-oviposition, whereas zygotic expression in embryos is known to start after 2 h [19].

Based on these data, it was expected that the fragment cloned would be able to drive the expression of an effector molecule in the same fashion as the fluorescent marker; however, when tTA replaced mNG, the transgenic lines obtained either failed to exhibit maternal expression, or it was detected only at very low levels (in the case of bZip-tTA-mNG F1). Only zygotic expression was detected in transgenic bZip-tTA lines, with even lower transcript abundance than observed for transgenic bZip-mNG lines in early embryos. These data, in addition to inverse PCR results, that confirmed different genomic position insertion for each line and the absence of the marker gene in the ovaries of bZip-tTA-mNG F1 line, indicate that chromosomal position can strongly influence expression from the *bZip1* fragment used. Less clear is why only poorly expressing strains were obtained from the bZip-tTA experiments. It is possible that this is simply due to chance, and that with additional independent insertions some would be found with more suitable positions for expression. Another possibility could be related to potential toxicity of the tTA proteins when expressed at high levels, thus dooming any such integrations to be lost from our experiments before they could be identified. tTA is known to be a toxic molecule as its expression was used in the transgenic line OX513A and its accumulation is cytotoxic leading the organism death [40, 41]. In addition, a report that used the pBac transposable element to generate an *An. stephensi* transgenic line with a repressible female-specific phenotype (fsRIDL) controlled by the Tet-off system, revealed an unusual single transgene integration [42]. pBac is known to generate multiples insertion [43, 44] and the authors discussed that the high expression of tTA caused by multiples copies of the transgene might not be tolerated and be deleterious as well as if integrated at a chromosomal environment that enhances expression. That observation might support why only lines with the lack of maternal expression and very low zygotic expression could actually be obtained in ours experiments as the tTA expression in germline tissues and/or the embryos might not be tolerated.

## Conclusions

In conclusion, a novel female germline and early zygote promoter from the transcription factor *AabZip1* was characterized and its potential use to express heterologous proteins evaluated. We successfully generated *Ae. aegypti* transgenic lines in which the *AabZip1* promoter expresses a fluorescent marker protein with the same pattern of expression as the endogenous gene. However, the genomic fragment chosen appeared to be strongly repressed by position effects and the choice of the molecule to be expressed should be taken in consideration when using transgenic approach for vector control. In particular, caution should be taken when using a molecule such as tTA with potential toxic effects as its expression in transgenic lines may be restricted.

## Supplementary information

**Additional file 1: Table S1.** List of primers. **Table S2.** AabZip1 has single reciprocal best-match orthologs in other mosquitoes but not in Drosophila. **Table S3.** AabZip1 has single reciprocal best-match orthologs in fleas and midges. **Figure S1**. Phylogenetic analysis of AabZip1 protein and its closest orthologs. **Figure S2**. Accumulation of mNG in ovaries of bZip-mNG F1 and F2 transgenic lines.

## Data Availability

All datasets generated or analyzed during the present study are presented within the article and its additional file.
