# Supplementary material for: Characterization of a female germline and early zygote promoter from the transcription factor bZip1 in the dengue mosquito Aedes aegypti
Source: Parasit Vectors. 2020 Jul 17;13:353. doi: 10.1186/s13071-020-04216-w (PMC7367395; doi:10.1186/s13071-020-04216-w)
Supplement: Supplementary file 1 — Additional file 1: Table S1. List of primers. Table S2. AabZip1 has single reciprocal best-match orthologs in other mosquitoes but not in Drosophila. Table S3. AabZip1 has single reciprocal best-match orthologs in fleas and midges. Figure S1. Phylogenetic analysis of AabZip1 protein and its closest orthologs. Figure S2. Accumulation of mNG in ovaries of bZip-mNG F1 and F2 transgenic lines. [file 13071_2020_4216_MOESM1_ESM.docx]

**Additional file 1: Table S1. List of Primers**

| **Inverse PCR (Adelman *et al*., 2007)** | |
| --- | --- |
| MLF1 | 5′-TTGTTTACTCTCAGTGCAGTCAACATGTCG-3′ |
| MLR1 | 5′-TTCGACAGTCAAGGTTGACACTTCACAAGG-3′ |
| MRF1 | 5′-AAGACGATGAGTTCTACTGGCGTGGAATCC-3′ |
| MRR1 | 5′-CTTGCCGTATGTGATGGAGCGTTGTCATGG-3′ |
| **qRT-PCR** | |
| mNG FO | 5′-AAGGGCGAGGAGGATAACAT-3′ |
| mNG REV | 5′-GATGGAAGCCATACCCGATA-3′ |
| bZip FO | 5′-AACCCACTGTGCATGAATGA-3′ |
| bZip REV | 5′-TCGTTCCTTGTGTTGAGCAG-3′ |
| tTA FO | 5′-GCCGCCATTATTACGACAAG-3′ |
| tTA REV | 5′-TGATCAATTCAAGGCCGAAT-3′ |
| qS7_F | 5′-accgccgtctacgatgcca-3′ |
| qS7_R | 5′-atggtggtctgctggttctt-3′ |

**Additional file 1: Table S2. AabZip1 has single reciprocal best match orthologs in other mosquitoes but not in *Drosophila*.**

|  | **Aln Length** | **E-value** | **Score** | **% Identity** |
| --- | --- | --- | --- | --- |
| **Query: AAEL009263 (AabZip1)** | |  |  |  |
| ***Aedes aegypti*** |  |  |  |  |
| AAEL009263-PA^a^ | 336 | 0 | 1813 | 100% |
| AAEL009263-PB^a^ | 336 | 0 | 1813 | 100% |
|  |  |  |  |  |
| ***Anopheles gambiae*** |  |  |  |  |
| AGAP007767-PA^b^ | 163 | 2.00E-27 | 275 | 38.80% |
| AGAP029153-PA^b^ | 79 | 0.004 | 88 | 31.70% |
| AGAP006461-PA^b^ | 102 | 0.007 | 86 | 24.30% |
|  |  |  |  |  |
| ***Culex quinquefasciatus*** |  |  |  |  |
| CPIJ000468-PA^c^ | 294 | 5.00E-72 | 586 | 45.70% |
| CPIJ001396-PA^c^ | 58 | 0.69 | 69 | 34.40% |
|  |  |  |  |  |
| ***Drosophila melanogaster*** |  |  |  |  |
| CG8420-PA^d^ | 34 | 9.52313 | 28.4906 | 58.80% |
|  |  |  |  |  |
| **Query: AGAP007767 (AngabZIP1)** | |  |  |  |
| ***Aedes aegypti*** |  |  |  |  |
| AAEL009263-PA | 160 | 9.00E-27 | 273 | 38.40% |
| AAEL009263-PB | 160 | 9.00E-27 | 273 | 38.40% |
| AAEL020773-PA | 61 | 0.3 | 73 | 32.30% |
|  |  |  |  |  |
| ***Anopheles gambiae*** |  |  |  |  |
| AGAP007767-PA | 368 | 0 | 1973 | 100% |
| AGAP004521-PA | 93 | 0.33 | 72 | 26.30% |
| AGAP004970-PA | 50 | 1.6 | 66 | 34.60% |
|  |  |  |  |  |
| ***Culex quinquefasciatus*** |  |  |  |  |
| CPIJ000468-PA | 161 | 7.00E-28 | 279 | 39.60% |
| CPIJ011252-PA | 61 | 0.085 | 77 | 33.90% |
| CPIJ019241-PA | 59 | 0.093 | 77 | 35% |
|  |  |  |  |  |
| ***Drosophila melanogaster*** |  |  |  |  |
| Xrp1-PA | 19 | 31.5722 | 1.39532 | 84% |

BLASTP search of Vectorbase or Flybase; ^a^AaegL5.2 geneset.2, ^b^AgamP4.12 geneset.10, ^c^CpipJ2.4 geneset.5, ^d^Dmel.r6.32

**Additional file 1: Table S3. AabZip1 has single reciprocal best match orthologs in fleas and midges**^#^

|  | Max Score | Total Score | Query Cover | E value | Per. Ident | Accession |
| --- | --- | --- | --- | --- | --- | --- |
| Query: AAEL009263 (AabZIP1) |  |  |  |  |  |  |
| uncharacterized protein LOC5571736 [Aedes aegypti] | 702 | 702 | 100% | 0 | 100.00% | XP_001659875.1 |
| AAEL009263-PB [Aedes aegypti] | 476 | 476 | 67% | 2.00E-167 | 100.00% | EAT38886.1 |
| uncharacterized protein LOC109411250 isoform X1 [Aedes albopictus] | 333 | 333 | 95% | 5.00E-110 | 54.57% | XP_019540312.2 |
| hypothetical protein RP20_CCG023752 [Aedes albopictus] | 328 | 328 | 95% | 1.00E-107 | 53.96% | KXJ80715.1 |
| uncharacterized protein LOC109411250 isoform X2 [Aedes albopictus] | 303 | 303 | 82% | 1.00E-98 | 55.63% | XP_019540313.2 |
| conserved hypothetical protein [Culex quinquefasciatus] | 230 | 230 | 87% | 2.00E-69 | 45.67% | XP_001842138.1 |
| bZIP1 [Anopheles albimanus] | 128 | 128 | 47% | 1.00E-29 | 43.12% | AFF19517.1 |
| AGAP007767-PA [Anopheles gambiae str. PEST] | 110 | 110 | 48% | 1.00E-23 | 38.79% | XP_317748.2 |
| AGAP007767-PA-like protein [Anopheles sinensis] | 110 | 110 | 47% | 2.00E-23 | 39.13% | KFB46204.1 |
| bZIP1 [Anopheles stephensi] | 100 | 100 | 48% | 4.00E-20 | 35.15% | AFF19515.1 |
| uncharacterized protein LOC113363354 [Ctenocephalides felis] | 80.1 | 80.1 | 46% | 3.00E-13 | 34.39% | XP_026461631.1 |
| uncharacterized protein LOC113386708 [Ctenocephalides felis] | 79.3 | 79.3 | 46% | 9.00E-13 | 33.76% | XP_026480256.1 |
| CLUMA_CG003455, isoform A [Clunio marinus] | 53.1 | 53.1 | 22% | 5.00E-04 | 36.84% | CRK89742.1 |
|  |  |  |  |  |  |  |
| Query: LOC113386708 [C felis] |  |  |  |  |  |  |
| uncharacterized protein LOC113386708 [Ctenocephalides felis] | 678 | 678 | 100% | 0 | 100.00% | XP_026480256.1 |
| uncharacterized protein LOC113363354 [Ctenocephalides felis] | 592 | 592 | 87% | 0 | 100.00% | XP_026461631.1 |
| AGAP007767-PA-like protein [Anopheles sinensis] | 90.5 | 90.5 | 59% | 2.00E-16 | 33.49% | KFB46204.1 |
| bZIP1 [Anopheles albimanus] | 88.6 | 88.6 | 36% | 1.00E-15 | 40.34% | AFF19517.1 |
| bZIP1 [Anopheles stephensi] | 80.9 | 80.9 | 35% | 3.00E-13 | 41.03% | AFF19515.1 |
| uncharacterized protein LOC5571736 [Aedes aegypti] | 79.3 | 79.3 | 45% | 1.00E-12 | 33.76% | XP_001659875.1 |
| AGAP007767-PA [Anopheles gambiae str. PEST] | 78.6 | 78.6 | 37% | 2.00E-12 | 37.40% | XP_317748.2 |
| uncharacterized protein LOC109411250 isoform X2 [Aedes albopictus] | 73.2 | 73.2 | 38% | 9.00E-11 | 28.35% | XP_019540313.2 |
| uncharacterized protein LOC109411250 isoform X1 [Aedes albopictus] | 73.2 | 73.2 | 38% | 1.00E-10 | 28.35% | XP_019540312.2 |
| hypothetical protein RP20_CCG023752 [Aedes albopictus] | 73.2 | 73.2 | 38% | 1.00E-10 | 28.35% | KXJ80715.1 |
| conserved hypothetical protein [Culex quinquefasciatus] | 62.4 | 62.4 | 28% | 5.00E-07 | 37.23% | XP_001842138.1 |
| CLUMA_CG003455, isoform A [Clunio marinus] | 57 | 57 | 26% | 3.00E-05 | 38.64% | CRK89742.1 |
|  |  |  |  |  |  |  |
| Query: CLUMA_CG003455 [C. marinus] |  |  |  |  |  |  |
| CLUMA_CG003455, isoform A [Clunio marinus] | 642 | 642 | 100% | 0 | 100.00% | CRK89742.1 |
| bZIP1 [Anopheles albimanus] | 68.9 | 68.9 | 36% | 4.00E-09 | 36.97% | AFF19517.1 |
| AGAP007767-PA [Anopheles gambiae str. PEST] | 67.4 | 67.4 | 24% | 1.00E-08 | 45.45% | XP_317748.2 |
| AGAP007767-PA-like protein [Anopheles sinensis] | 65.5 | 65.5 | 24% | 5.00E-08 | 44.16% | KFB46204.1 |
| CLUMA_CG003461, isoform A [Clunio marinus] | 65.1 | 65.1 | 36% | 6.00E-08 | 31.62% | CRK89739.1 |
| bZIP1 [Anopheles stephensi] | 61.2 | 61.2 | 34% | 1.00E-06 | 38.53% | AFF19515.1 |
| uncharacterized protein LOC113363354 [Ctenocephalides felis] | 57 | 57 | 27% | 2.00E-05 | 38.64% | XP_026461631.1 |
| uncharacterized protein LOC113386708 [Ctenocephalides felis] | 57 | 57 | 27% | 3.00E-05 | 38.64% | XP_026480256.1 |
| uncharacterized protein LOC5571736 [Aedes aegypti] | 53.1 | 53.1 | 23% | 5.00E-04 | 36.84% | XP_001659875.1 |
| uncharacterized protein LOC109411250 isoform X2 [Aedes albopictus] | 49.3 | 49.3 | 24% | 0.008 | 35.90% | XP_019540313.2 |
| uncharacterized protein LOC109411250 isoform X1 [Aedes albopictus] | 49.3 | 49.3 | 24% | 0.009 | 35.90% | XP_019540312.2 |
| hypothetical protein RP20_CCG023752 [Aedes albopictus] | 49.3 | 49.3 | 24% | 0.01 | 35.90% | KXJ80715.1 |
| conserved hypothetical protein [Culex quinquefasciatus] | 48.5 | 48.5 | 23% | 0.018 | 38.16% | XP_001842138.1 |

^#^BLASTP search of NCBI NR database (Feb 2020)


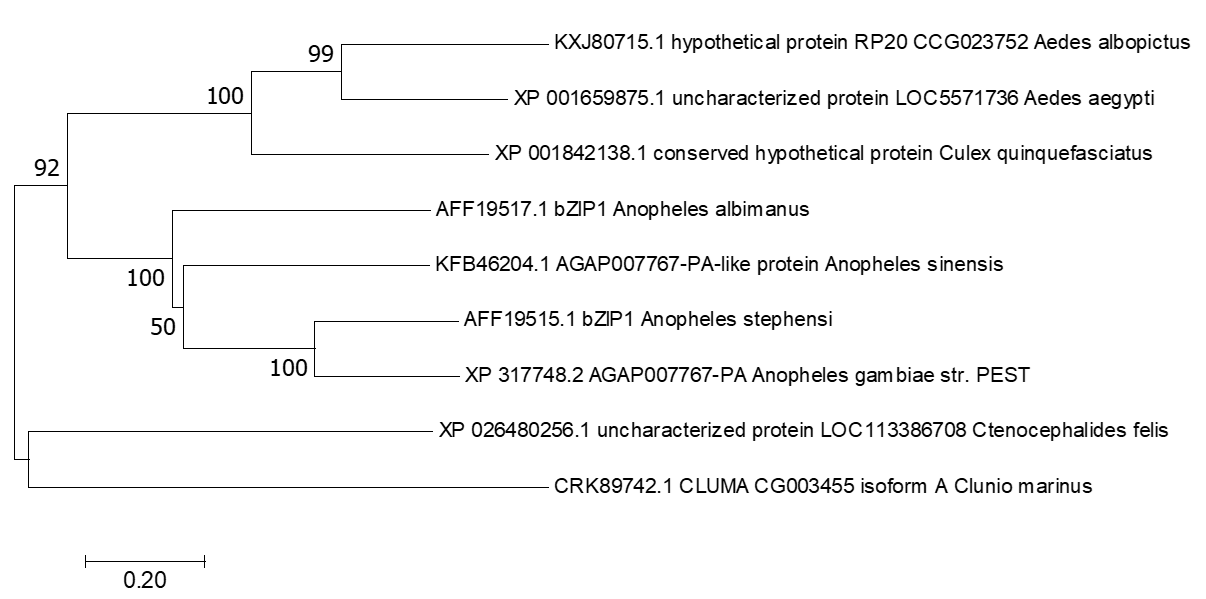


**Additional file 1: Figure S1. Phylogenetic analysis of AabZip1 protein and its closest orthologs.** Neighbor-joining tree constructed following alignment using Muscle as implemented by MEGA 7 (reference) using the Poisson correction method at 1000 bootstrap replicates. The percent bootstrap support is indicated when >50%.

**
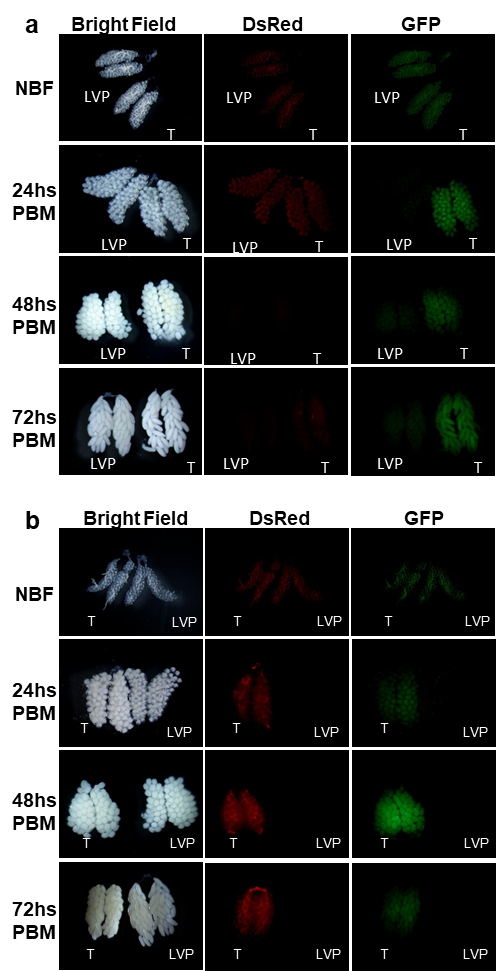
**

**Additional file 1: Figure S2.** Accumulation of mNG in ovaries of bZip-mNG F1 and F2 transgenic lines. Ovaries from bZip-mNG F1 (a) and F2 (b) transgenic lines (T) and wild type (LVP) at 24, 48 and 72h after a blood meal.
